# Choice of Surgical Technique in Groin Hernia Surgery Among Residents in Senegal: Experience and Influencing Factors

**DOI:** 10.3389/jaws.2025.14076

**Published:** 2025-05-30

**Authors:** Abdourahmane Ndong, Adja Coumba Diallo, Adebayo Falola, Mamadou Arame Ndiaye, Magatte Faye, Pape Mamadou Faye, Abdou Niasse, Sidy Mouhamed Abdoulaye Fall, Mamadou Cissé, Papa Saloum Diop, Ibrahima Konaté

**Affiliations:** ^1^ Department of Surgery, Saint-Louis Regional Hospital, Gaston Berger University, Saint-Louis, Senegal; ^2^ Department of Medicine and Surgery, University of Ibadan College of Medicine, Ibadan, Nigeria; ^3^ Department of Surgery, Hôpital Principal, Dakar, Senegal; ^4^ Department of Surgery, Cheikh Anta Diop University, Dakar, Senegal; ^5^ General Surgery Unit of Cheikh Ahmadoul Khadim Hospital in Touba, Alioune Diop University Of Bambey, Diourbel, Senegal; ^6^ Centre Hospitalier Regional Heinrich Lübke, Diourbel, Senegal

**Keywords:** hernia, surgery, Bassini, laparoscopy, Africa

## Abstract

**Introduction:**

Groin hernia repair is a common surgical procedure globally, with several techniques developed to minimize complications, pain, and recurrence. In Senegal, despite advancements, the adoption of minimally invasive techniques remains limited. This study evaluates the factors influencing surgical technique choice among surgical residents in Senegal.

**Methods:**

We conducted a national cross-sectional survey from July 1 to July 15, 2024. The survey targeted surgical residents in Senegal specializing in general surgery and urology, utilizing contact information from resident associations. A structured questionnaire covered demographic data, knowledge and experience with hernia repair techniques, and factors influencing technique choice. Statistical analysis was performed using R software, with descriptive statistics summarizing the data.

**Results:**

A total of 74 residents participated, with an average age of 31.3 ± 3.5 years. The Bassini repair was the most commonly known (100%), witnessed (97.3%), and performed technique (94.2%) among residents, followed by the Lichtenstein technique. Minimally invasive techniques, such as TAPP and TEP, were less familiar and rarely performed. Factors influencing technique choice included training (90.5%), ease of performance (63.5%), time required (58.1%), and cost (51.4%).

**Conclusion:**

This study highlights a preference for open hernia repair techniques among residents in Senegal, particularly the Bassini and Lichtenstein techniques, due to cost-effectiveness and accessibility. Limited training and resources constrain the adoption of minimally invasive techniques, underscoring the need for enhanced access to resources and training to align with global standards.

## Introduction

Hernias, characterized by the protrusion of organs or tissues through openings in the abdominal wall, are a common condition worldwide [1]. In Africa, it accounts for one of the major general surgical procedures performed [2]. Over time, hernia repair surgery has undergone significant advancements, leading to the development of various techniques aimed at reducing postoperative complications, pain, and recurrence [3]. These innovations also focus on accelerating patient recovery, improving quality of life, and minimizing both post-surgical discomfort and potential adverse effects [3, 4]. The ideal surgical technique, however, remains a topic of debate. Various methods feature distinct technical differences, such as the surgical approach, types of mesh used, methods or use of mesh fixation, and the management of the hernia sac [5, 6].

Conventional techniques include the Bassini repair, one of the earliest methods for open hernia repair [7]. The Lichtenstein repair, introduced in 1984, is a tension-free approach designed to eliminate the need for tension sutures used in earlier techniques [8, 9]. The Desarda technique, which is a non-mesh and tension-free repair, is associated with shorter surgery duration and reduced rates of hernia recurrence [10]. The Shouldice repair is known for its meticulous anatomical precision and low recurrence rates [11]. McVay repairs are primarily used for treating femoral hernias [12]. Minimally invasive hernia repair methods, such as Trans Abdominal PrePeritoneal (TAPP) and Totally Extra Peritoneal (TEP), offer advantages over conventional open surgery [13]. In sub-Saharan Africa, particularly in Senegal, the adoption of these minimally invasive approaches has however been slow and gradual [14]. To date, there have only been two reports on the use of laparoscopy for groin hernia repair in Senegal [14], and the robotic approach is yet to be introduced [15].

The decision on which technique to use for hernia repair is usually not made independently, but determined by resource availability, protocols of the institution, and the supervising surgeons [1]. This study aims to evaluate the experience and factors influencing the choice of surgical techniques for groin hernia surgery among surgical residents in Senegal. Our findings will contribute to the development of tailored surgical training and strategies in hernia surgery for residents in Senegal.

## Methodology

The CHERRIES (Checklist for Reporting Results of Internet E-Surveys) guidelines were followed for this study [16].

### Design

This was a national cross-sectional survey. The study period was from July 1 to July 15, 2024.

### Population and Sampling

The target population included all surgical residents currently in training in Senegal. Only specialties dealing with groin hernia were included (general surgery and urology). Contact information (phone numbers and/or email addresses) was obtained from Senegalese resident associations (AIAIHS and COMES). Four cycles of emails and messages were sent to increase the response rate.

### Study Setting

The study was conducted in Senegal, a West African country with an estimated population of 18,032,473 in 2023. There are 5 public universities in Senegal, of which 3 have surgical residency programs. In Senegal, groin hernia is managed by general surgeons and urologists.

### Survey Tool and Studied Parameters

A structured questionnaire (added as Supplementary Material) was designed for the study to collect data on factors influencing the choice of surgical technique in groin hernia repair. The questionnaire was pre-tested to ensure clarity and relevance. The survey was designed to be concise and easy to complete. The questions were divided into 3 sections:

• Section I: focused on demographic data including specialty, university, year of specialization, sex, and age.
• Section II: concentrated on knowledge and experience, including known surgical techniques for hernia repair, techniques witnessed or assisted with, experience as primary operator, and number of groin hernia repairs performed during training.
• Section III: addressed preferences and influencing factors, concerning their preferred surgical technique for uncomplicated inguinal hernia in young men, factors influencing technique choice (training, ease of performance, duration, cost, complications).

### Data Analysis

Qualitative variables in the descriptive study were described in terms of frequency and proportion, while quantitative variables were presented as means with their extremes or standard deviations. Graphs were generated with Microsoft Excel.

### Consent

All participants were asked for consent before participation, and all data were anonymized.

### Ethical Considerations

The study protocol was approved by the Institutional Review Board. No personal identifying information was collected to ensure anonymity.

### Statistical Analysis

Data were analyzed using R software. Descriptive statistics were used to summarize demographic data and responses.

## Results

### Demographics

A total of 74 residents participated in this study, with a mean age of 31.3 ± 3.5 years. Most participants were male (86.5%, n = 64), while females constituted 13.5% (n = 10) of the sample. General surgery residents accounted for 59.5% (n = 44) of the participants, with the remaining 40.5% (n = 30) being urology residents.

Regarding institutional affiliation, 51.4% (n = 38) of the residents were from Cheikh Anta Diop University, 35.1% (n = 26) from Gaston Berger University, and 13.5% (n = 10) from the University of Thiès. The distribution across years of residency was relatively uniform, with 25.7% (n = 19) in their first year, 20.3% (n = 15) each in their second and third years, 18.9% (n = 14) in their fourth year, and 14.9% (n = 11) in their fifth year. These characteristics are described in Table 1.

**TABLE 1:** Baseline characteristics of patients.

Variable (n = 74)	Categories	N(%) or Mean standard ± deviation
Speciality (%)	General surgery	44 (59.5)
	Urology	30 (40.5)
University (%)	Cheikh Anta Diop University	38 (51.4)
	Gaston Berger University	26 (35.1)
	University of Thiès	10 (13.5)
Year of residency (%)	1st year	19 (25.7)
	2nd year	15 (20.3)
	3rd year	15 (20.3)
	4th year	14 (18.9)
	5th year	11 (14.9)
Sex (%)	Female	10 (13.5)
	Male	64 (86.5)
Age		31.3 ± 3.5

### Knowledge and Experience

Knowledge of surgical techniques for hernia repair varied among the residents. The most widely known techniques was Bassini repair (100%, n = 74), Lichtenstein repair (91.9%, n = 68), followed by Desarda (85.1%, n = 63), McVay (75.7%, n = 56), and Shouldice (66.2%, n = 49). Laparoscopic techniques such as TAPP (Transabdominal Preperitoneal) and TEP (Totally Extra Peritoneal) were less commonly known, with 44.6% (n = 33) and 37.8% (n = 28) of residents familiar with these techniques, respectively.

**FIGURE 1:**
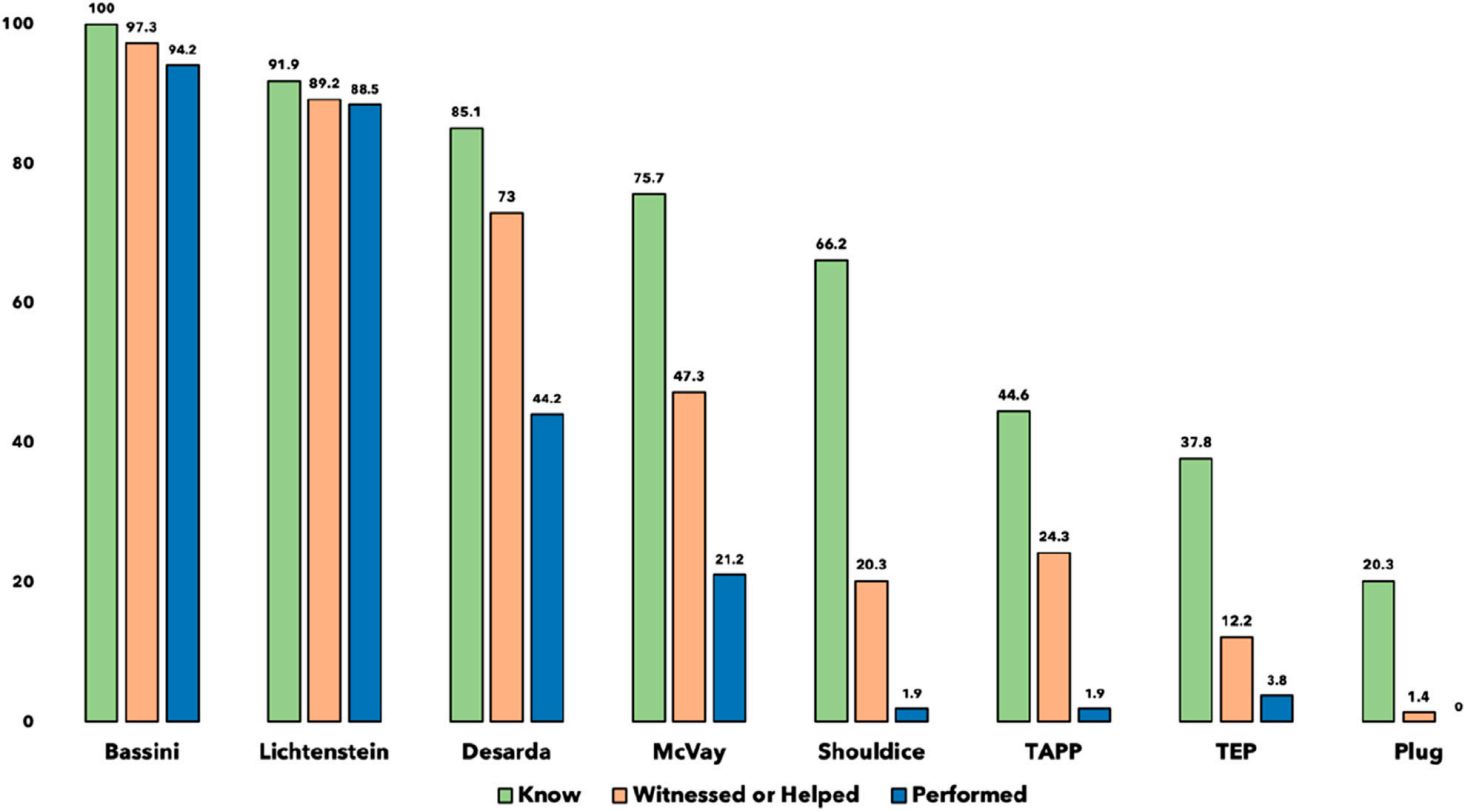
Knowledge, experience, and performance of groin hernia repair techniques among surgical residents (n = 74).

Regarding practical experience, the Bassini technique was the most commonly witnessed or assisted procedure (97.3%, n = 72), followed by Lichtenstein (89.2%, n = 66) and Desarda (73.0%, n = 54). In contrast, minimally invasive techniques such as TAPP (24.3%, n = 18) and TEP (12.2%, n = 9) were less frequently observed or assisted. Figure 1 describes knowledge, experience, and performance of groin hernia repair techniques among surgical residents.

**FIGURE 2:**
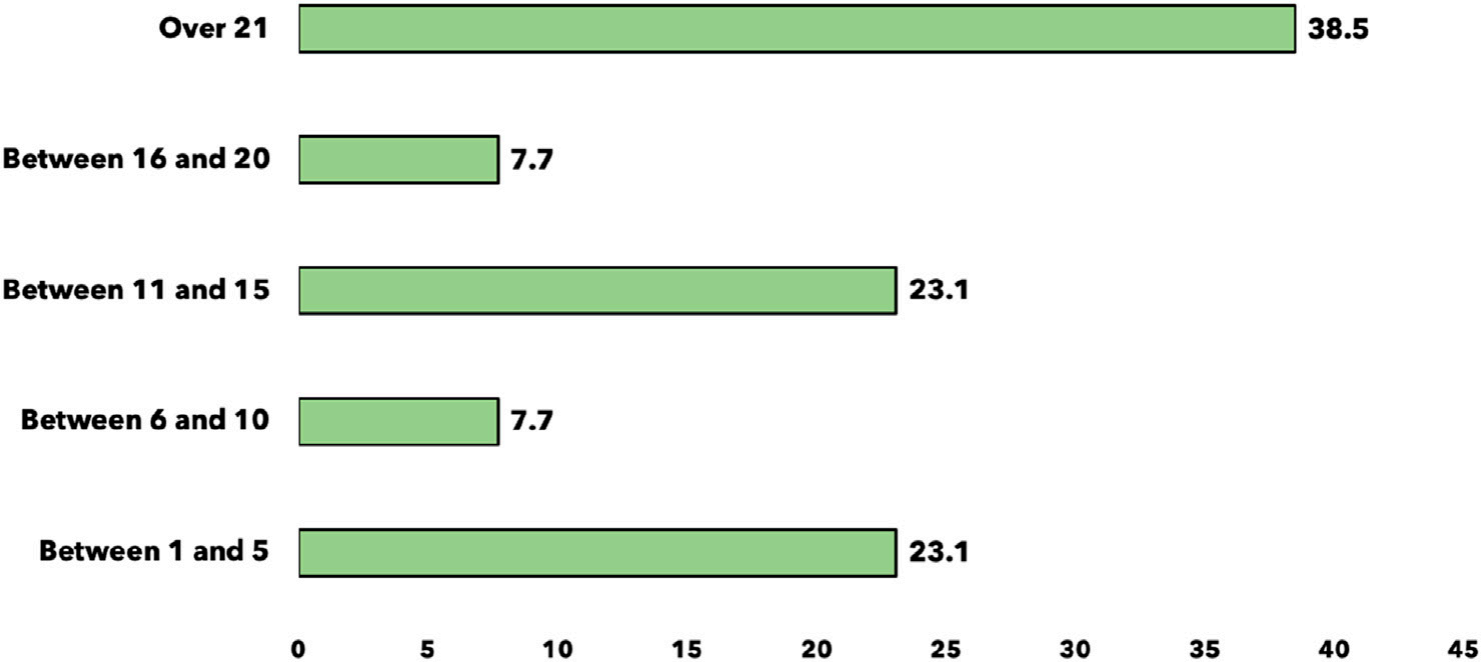
Number of groin hernia repairs performed as primary operator (n = 74).

Notably, 70.3% (n = 52) of the residents reported having performed a groin hernia repair as the primary surgeon (Figure 2). Among these, the most frequently performed techniques were Bassini (94.2%, n = 49) and Lichtenstein (88.5%, n = 46). Laparoscopic techniques were rarely performed, with only 1.9% (n = 1) reporting experience with TAPP and 3.8% (n = 2) with TEP (Figure 2).

**FIGURE 3:**
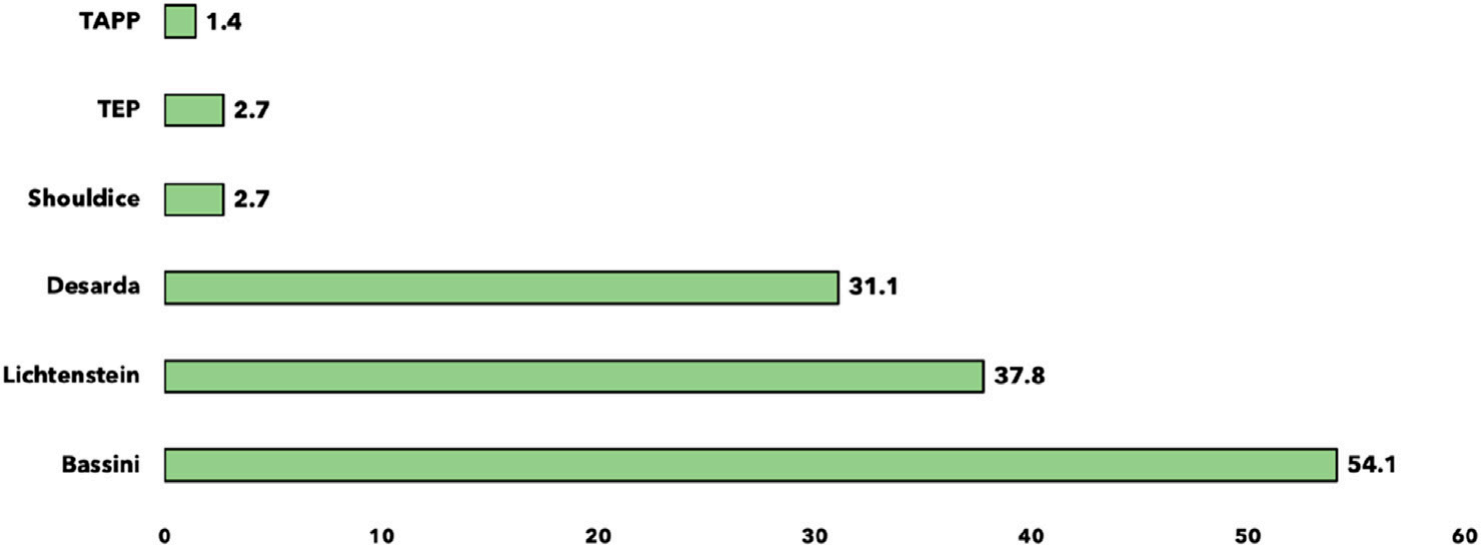
Preferred surgical techniques for young men with uncomplicated inguinal hernia (n = 74).

The number of groin hernia repairs performed by residents as primary surgeons varied, with 38.5% (n = 20) reporting over 21 procedures, while 23.1% (n = 12) had performed between 1 and 5 repairs (Figure 3).

### Preferences and Influencing Factors

**FIGURE 4:**
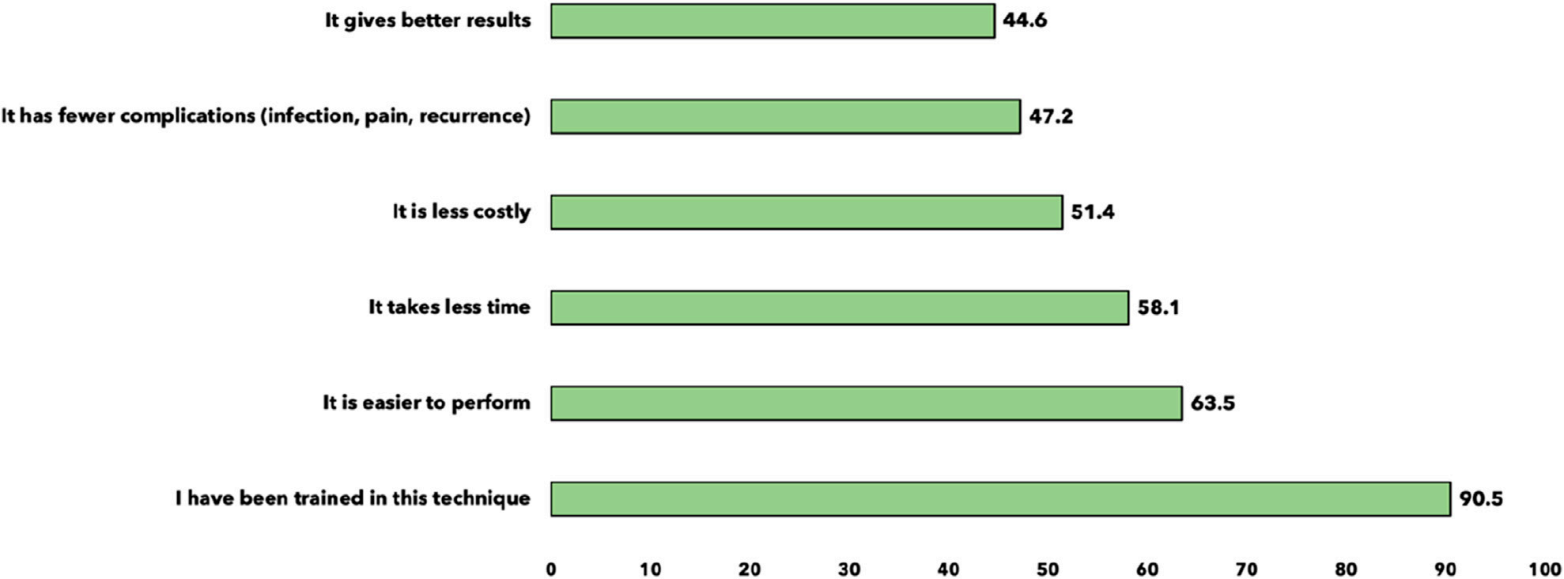
Factors influencing the choice of surgical technique for groin hernia repair (n = 74).

When asked about their preferred surgical technique for a young man with an uncomplicated inguinal hernia, the Bassini technique was the most popular choice (54.1%, n = 40), followed by Lichtenstein (37.8%, n = 28), and Desarda (31.1%, n = 23). Minimally invasive techniques such as TAPP and TEP were rarely preferred, chosen by only 1.4% (n = 1) and 2.7% (n = 2) of residents, respectively (Figure 3). The factors influencing the choice of surgical technique for inguinal hernia surgery among residents were the training on a technique in 90.5%, ease of performance in 63.5%, time required in 58.1%, cost in 51.4%, complications (infection, pain, recurrence) in 47.2%, and clinical results in 44.6% (Figure 4).

## Discussion

Hernia repair is becoming an increasingly complex field with the introduction of new techniques, including minimally invasive approaches, and a significant rise in the variety of available mesh products [17]. As a result of these rapid advancements, the level of experience among residents in hernia repair varies widely [18–20]. Also, various factors such as available resources and training affect the choice of hernia repair [21]. However, mastering a range of techniques, from the simplest to the most advanced, is essential to achieve optimal surgical outcomes for patients [18]. Our survey aimed to assess the variation in hernia training among surgical residents in Senegal. With 74 residents across the three surgical residency programs in Senegal participating, this sample is representative of the general surgery and urology residents across the country.

This survey revealed several key results, particularly regarding the varying levels of knowledge and experience regarding surgical techniques among residents. The Bassini repair followed by Lichtenstein repair emerged as the most known procedures. This finding is similar to findings among surgical trainees in Kenya [21], but differs from surveys among surgeons in other settings [22, 23]. For example, 98% of the surgeons in a large-scale international survey preferred repair with the mesh [23]. Our finding aligns with the status of Bassini as a cost-effective and easy to learn technique [10], while Lichtenstein is also popular for being the standard treatment for adult inguinal hernia due to the relatively high recurrence rates associated with tissue-based repairs [3]. Globally, Lichtenstein repair remains the leading technique for anterior hernia repair [3]. Its popularity is attributed to its low recurrence and minimal complication rates [24]. While no single technique is suitable for all groin hernias, and understanding multiple approaches is essential, open surgery, particularly the Lichtenstein method, seems to be the most widely practiced and accepted technique worldwide [8]. This method meets many criteria for an ideal open repair, effectively addressing the public health burden of hernia by minimizing recurrence, and morbidity, making residents’ familiarity with this technique crucial [8].

Regarding practical experience, the Bassini technique was the most commonly witnessed or assisted procedure, followed by Lichtenstein and Desarda. While minimally invasive techniques such as TAPP and TEP were less frequently observed or assisted. Also, the most frequently performed techniques were Bassini and Lichtenstein, while laparoscopic techniques were rarely performed. While the Lichtenstein technique remains the standard for adult open inguinal hernia repair [3], the Shouldice method stands out among non-mesh repairs, demonstrating results comparable to mesh-based techniques in certain cases [25, 26]. Its popularity has been growing, partly due to concerns over chronic pain associated with the Lichtenstein approach [27, 28]. In our study, however, only 1.9% of the residents had performed Shouldice as the primary operator.

Our findings regarding the higher use of tissue-based repairs contrast practice in other settings. In a survey among the members of the British Hernia Society, for example, most of the surgeons preferred an open Lichtenstein repair, while the others would apply laparoscopic techniques, and 95% of the surgeons never performed an open tissue repair [22]. In sub-Saharan Africa, tissue-based repairs like the Shouldice and Bassini techniques are more frequently utilized due to the high costs associated with mesh, despite international guidelines recommending mesh-based repairs [2, 25]. The average cost of mesh is around $11, and the total expense of a mesh-based procedure is approximately $200, making it prohibitively expensive in low-income settings, including countries like Senegal [2, 29]. This explains the wider use and exposure of the residents to the tissue-based techniques in these regions.

In our study, the Bassini technique was the most commonly observed and performed by residents, despite the Shouldice technique being the preferred option in low-income settings [3]. This is largely due to the longer learning curve associated with Shouldice, whereas Bassini is both cost-effective and easier to learn [26]. The Lichtenstein technique was observed and performed by 89.2% and 88.5% of residents, respectively, indicating its continued widespread use in Senegal despite the higher costs involved [30, 31]. The Desarda technique was observed or assisted in 73.0% of cases and performed by 44.2% of residents. Several studies have found that the Desarda repair shows similar outcomes to Lichtenstein, Bassini, and laparoscopic repairs in terms of recurrence rates, chronic pain, complications, mobility, return to normal activities, and cost [24, 32]. The Desarda method also has a shorter learning curve than the Shouldice, the currently recommended technique in settings like Senegal [10, 33]. However, Desarda was less frequently encountered by residents, likely because it is often reserved for emergency hernia repairs due to concerns about the safety of mesh in emergency situations with infection risks, and because it is a tension-free repair compared to Lichtenstein and Shouldice [32]. Besides, since a large part of hernia is treated in emergency when strangulated with bowel obstruction, mesh based repairs are less frequently used in our context [34].

The TAPP and TEP minimally invasive techniques are associated with benefits such as reduced postoperative pain and quicker recovery times [13, 35]. Despite these advantages, they were the least performed and preferred techniques, likely due to the limited availability of laparoscopic equipment for training, driven by high costs [2, 36]. This is also a finding in other African countries [21]. Training on a technique is a major factor that influences the choice of repair since individual professional skill and proficiency is best expressed in such technique [21, 37]. For example, a survey done in Turkey showed that residents who had prior experience with laparoscopic hernia repair were more likely to choose the technique [38]. This was also observed in our study with 90.5% of the residents choosing a repair method because they were training on it. Although our study does not include data on the choice of technique for bilateral inguinal hernia repair, it is important to note that the minimally invasive approach is the recommended technique by international guidelines for managing bilateral inguinal hernia because it allows both groins to be addressed through the same incisions used for unilateral repair, making it a cost-effective option compared to open surgery [3, 4]. Regarding recurrent hernias, a laparoscopic posterior repair is preferred after a failed anterior repair, while an anterior approach is best after failed laparoscopic or posterior open repairs [3]. Robotic surgery is yet to be introduced in Senegal [15]. As a result, we did not collect data on residents’ knowledge and preferences regarding robotic hernia repair.

## Conclusion

This study describes factors influencing the choice of surgical techniques among surgical residents in Senegal for groin hernia repair. The findings show a predominant preference for open, tissue-based repairs, particularly the Bassini technique. This preference seems to be explained by practical considerations in resource-limited settings like the cost associated with mesh and endoscopic equipment; and the relatively straightforward learning curve of the Bassini method. However, Lichtenstein is the second most commonly observed and performed technique among residents. Notably, the Desarda technique is also used because it offers an affordable and feasible alternative when mesh access is limited. Conversely, the uptake of minimally invasive techniques, including TAPP and TEP, is minimal, primarily due to limited access to the necessary equipment and training resources. This fact explains why many residents remain less or untrained in these techniques. Overall, this study shows the need for targeted interventions to improve access to resources, such as mesh and laparoscopic equipment, and to enhance training in advanced hernia repair methods.

## Data Availability

The data supporting the findings of this study are available from the corresponding author upon reasonable request. Requests to access the datasets should be directed to Corresponding author.
