# Supplementary material for: Choice of Surgical Technique in Groin Hernia Surgery Among Residents in Senegal: Experience and Influencing Factors
Source: J Abdom Wall Surg. 2025 May 30;4:14076. doi: 10.3389/jaws.2025.14076 (PMC12162351; doi:10.3389/jaws.2025.14076)
Supplement: Supplementary file 1 [file DataSheet2.pdf]

Machine Translated by Google

QUIZ

Speciality

- ◯ Urology
- ◯ General surgery

University

- ◯ Cheikh Anta Diop University
- ◯ University of Thiès
- ◯ Gaston Berger University

Year of DES

- ◯ 1st year
- ◯ 2nd year
- ◯ 3rd year
- ◯ 4th year
- ◯ 5th year/Memory

Sex

- ◯ Female
- ◯ Male

Age

____

HERNIA

Hernia Repair Surgical Techniques You Know

You can check multiple options if necessary

- ☐ Shouldice
- ☐ Basin
- ☐ McVay
- ☐ Desarda
- ☐ Liechtenstein
- ☐ Plug
- ☐ TAPP
- ☐ TEP

Machine Translated by Google

Surgical hernia repair techniques that you have already witnessed, assisted with or been trained in

You can check multiple options if necessary

- ☐ Shouldice
- ☐ Basin
- ☐ McVay
- ☐ Desarda
- ☐ Liechtenstein
- ☐ Plug
- ☐ TAPP
- ☐ TEP

Have you ever performed a groin hernia repair as a primary operator?

- ○ Yes
- ○ No

If yes, what technique(s) did you use?

You can check multiple options if necessary

- ☐ Shouldice
- ☐ Basin
- ☐ McVay
- ☐ Desarda
- ☐ Liechtenstein
- ☐ Plug
- ☐ TAPP
- ☐ TEP

Number of groin hernia repairs performed during training as a lead operator

- ○ None
- ○ between 1 and 5
- ○ Between 6 and 10
- ○ Between 11 and 15
- ○ Between 16 and 20
- ○ Over 21

Machine Translated by Google

What is your preferred surgical technique for a young man with an uncomplicated inguinal hernia?

You can check multiple options if necessary

- ☐ Shouldice
- ☐ Basin
- ☐ McVay
- ☐ Desarda
- ☐ Liechtenstein
- ☐ Plug
- ☐ TAPP
- ☐ TEP

For what?

You can check multiple options if necessary

- ☐ I was trained in this technique
- ☐ It is easier to achieve
- ☐ It gives better results
- ☐ It takes less time
- ☐ It is less expensive
- ☐ It has fewer post-operative complications (infection, pain, recurrence)
- ☐ Others
