# Supplementary material for: Choice of Surgical Technique in Groin Hernia Surgery Among Residents in Senegal: Experience and Influencing Factors
Source: J Abdom Wall Surg. 2025 May 30;4:14076. doi: 10.3389/jaws.2025.14076 (PMC12162351; doi:10.3389/jaws.2025.14076)
Supplement: Supplementary file 2 [file DataSheet1.pdf]

QUESTIONNAIRE

Spécialité

- ◯ Urologie
- ◯ Chirurgie générale

Université

- ◯ Université Cheikh Anta Diop
- ◯ Université de Thiès
- ◯ Université Gaston Berger

Annee de DES

- ◯ 1ère annee
- ◯ 2ème annee
- ◯ 3ème annee
- ◯ 4ème annee
- ◯ 5ème annee/Mémoire

Sexe

- ◯ Féminin
- ◯ Masculin

Age

____

HERNIE

Techniques chirurgicales de cure de hernie que vous connaissez

Vous pouvez cocher plusieurs options si necessaire

- ☐ Shouldice
- ☐ Bassini
- ☐ Mc Vay
- ☐ Desarda
- ☐ Lichtenstein
- ☐ Plug
- ☐ TAPP
- ☐ TEP

Techniques chirurgicales de cure de hernies auxquelles vous avez déjà assisté, aidé ou sur lesquelles vous avez été formé

Vous pouvez cocher plusieurs options si necessaire

- [ ] Shouldice
- [ ] Bassini
- [ ] Mc Vay
- [ ] Desarda
- [ ] Lichtenstein
- [ ] Plug
- [ ] TAPP
- [ ] TEP

Avez-vous déjà réalisé en tant qu'opérateur principal une cure de hernie de l'aine?

- ◯ Oui
- ◯ Non

Si Oui, quel(les) techniques avez-vous utilisé?

Vous pouvez cocher plusieurs options si necessaire

- [ ] Shouldice
- [ ] Bassini
- [ ] Mc Vay
- [ ] Desarda
- [ ] Lichtenstein
- [ ] Plug
- [ ] TAPP
- [ ] TEP

Nombre de cures de hernie de l'aine réalisées durant la formation en tant qu'opérateur principal

- ◯ Aucun
- ◯ entre 1 et 5
- ◯ Entre 6 et 10
- ◯ Entre 11 et 15
- ◯ Entre 16 et 20
- ◯ Plus de 21

Quelle est votre technique chirurgicale préférée pour un jeune homme avec une hernie inguinale non compliquée ?

Vous pouvez cocher plusieurs options si necessaire

- [ ] Shouldice
- [ ] Bassini
- [ ] Mc Vay
- [ ] Desarda
- [ ] Lichtenstein
- [ ] Plug
- [ ] TAPP
- [ ] TEP

Pourquoi?

Vous pouvez cocher plusieurs options si necessaire

- [ ] J'ai été formé sur cette technique
- [ ] Elle est plus facile à réaliser
- [ ] Elle donne de meilleurs résultats
- [ ] Elle prend moins de temps
- [ ] Elle est moins coûteuse
- [ ] Elle a moins de complications post-opératoires (infection, douleur, récidive)
- [ ] Autres
